# Performance of in-hospital mortality prediction models for acute hospitalization: Hospital Standardized Mortality Ratio in Japan

**DOI:** 10.1186/1472-6963-8-229

**Published:** 2008-11-07

**Authors:** Hiroaki Miyata, Hideki Hashimoto, Hiromasa Horiguchi, Shinya Matsuda, Noboru Motomura, Shinichi Takamoto

**Affiliations:** 1 Department of Healthcare Quality Assessment, Graduate School of Medicine, University of Tokyo, Japan; 2 Department of Health Economics and Epidemiology Research, School of Public Health, University of Tokyo, Japan; 3 Department of Health Management and Policy, Graduate School of Medicine, University of Tokyo, Japan; 4 Department of Preventive Medicine and Community Health, University of Occupational and Environmental Health, Fukuoka, Japan; 5 Department of Cardiothoracic Surgery, Faculty of Medicine, University of Tokyo, Japan

## Abstract

**Objective:**

In-hospital mortality is an important performance measure for quality improvement, although it requires proper risk adjustment. We set out to develop in-hospital mortality prediction models for acute hospitalization using a nation-wide electronic administrative record system in Japan.

**Methods:**

Administrative records of 224,207 patients (patients discharged from 82 hospitals in Japan between July 1, 2002 and October 31, 2002) were randomly split into preliminary (179,156 records) and test (45,051 records) groups. Study variables included Major Diagnostic Category, age, gender, ambulance use, admission status, length of hospital stay, comorbidity, and in-hospital mortality. ICD-10 codes were converted to calculate comorbidity scores based on Quan's methodology. Multivariate logistic regression analysis was then performed using in-hospital mortality as a dependent variable. C-indexes were calculated across risk groups in order to evaluate model performances.

**Results:**

In-hospital mortality rates were 2.68% and 2.76% for the preliminary and test datasets, respectively. C-index values were 0.869 for the model that excluded length of stay and 0.841 for the model that included length of stay.

**Conclusion:**

Risk models developed in this study included a set of variables easily accessible from administrative data, and still successfully exhibited a high degree of prediction accuracy. These models can be used to estimate in-hospital mortality rates of various diagnoses and procedures.

## Background

Numerous studies have shown that the quality of healthcare is variable and often inadequate [1-3]. Initiatives to measure healthcare quality are an important focus for policymakers who believe that such measurements can drive quality-improvement programs [4]. The measurement of healthcare quality includes process and outcome measurements [5]. Outcome evaluation, including in-hospital mortality, requires adequate risk-adjustment for different patient mixes to make appropriate evaluations of healthcare performance [6]. Because of the clear definition of outcome and influential patient conditions, disease-specific risk adjustment models have been developed to a certain extent in several specialties (e.g. cardiovascular diseases) and have been available for various quality improvement activities [7-10].

Although disease-specific risk adjustment may be useful for quality improvement of a specific type of care, more generic case-mix risk-standardized outcomes are required for generalized quality evaluation across specialties [11]. In the United States, several generic case-mix measures are available in commercial as well as non-commercial sources (e.g. APACHE, MedisGroup, Adjusted Clinical Group, Diagnostic Cost Groups, and the RxRisk model) [12-14], and have been applied to categorizing patients according to resource needs. However, many of these systems require detailed clinical and/or administrative data that involve extensive data collection. Furthermore, most of these case-mix measures target healthcare costs rather than clinical outcomes.

To alleviate the burden of data collection, risk prediction models for in-hospital mortality using administrative data have been proposed [15,27-29]. One study used a modified version of the Charlson Index [16] as a summary score of co-existing diagnoses. A recent international comparative study [17] demonstrated that the estimated comorbidity index could predict the chance of in-hospital death with relatively high precision (c-index of approximately 0.80), although the accuracy was suboptimal when Japanese data were analyzed. In this study, we developed a new prediction model for in-hospital mortality by using the same electronic dataset with national standardized format used in the aforementioned study. We successfully exceeded previously demonstrated predictive precision by including patient demographics and multiple administrative variables. Our study demonstrates a potential use of the developed prediction model for benchmarking the quality of healthcare across various performance units with the national database.

## Methods

### Data source

We used a dataset provided by the Ministry of Health, Labor, and Welfare that was originally used to evaluate a patient classification system newly introduced to 80 university affiliated hospitals and 2 national center hospitals for reimbursement since 2003. The new classification system, called Diagnosis Procedure Combination (DPC), includes information regarding up to two major diagnoses and up to six co-existing diagnoses. The 2003 version of the DPC patient classification system includes 16 major diagnosis categories (MDC) and 575 disease subcategories which are coded in ICD-10 format. The dataset also included additional information on patient demographics, use and types of surgical procedures, emergency/elective hospitalization, length of stay (LOS), and discharge status including in-hospital death [18-20]. The dataset originally included information derived from hospital administrative and clinical information provided by participating hospitals to the Ministry research group, then was made anonymous and fed back to the hospitals for benchmarking purposes. Records for 282,064 patients who were discharged from 82 hospitals between July 1, 2002 and October 31, 2002 were distributed and made available for public use as of June 2008. Following the inclusion criteria of previous studies on Hospital Standardized Mortality Ratio (HSMR) [21,22], we excluded MDC categories with mortality rates of less than 0.5% from our analysis. The data (n = 224,207) were then randomly assigned further into two subsets that were split 80/20, one for model development and the other for validation tests. The development dataset included 179,156 records and the validation dataset included 45,051 records. The datasets were made anonymous and prepared by the government sector for public use. Thus, data use was officially approved and protection of confidential information is ensured.

### Model building

We started with a prediction model by referring to the Canadian model of HSMR as mentioned earlier [21,22]. The model includes age as the ordinal variable (under 60, 60–69, 70–79, 80–89, and 90 and over), gender, use of an ambulance at admission, admission status (emergency/elective), LOS, MDC, and comorbidities (model 1). We also tested another prediction model which omitted LOS (model 2). The rationale is that the model without LOS should be a "pure" prediction model since LOS can be regarded as an outcome affected by patient characteristics and hospital care quality. Several diagnosis-specific models also consider the duration of hospitalization as a part of outcome and do not include it as a predictor variable [23,24]. Based on Quan's methodology [15], the ICD-10 code of each co-existing diagnosis was converted into a score, and was summed up for each patient case to calculate a Charlson Comorbidity Index score. Scores were then classified into five categories: 0, 1–2, 3–6, 7–12, and 13 and over.

We did not include surgical treatment status as a risk parameter because the decision of whether or not to operate on a patient with a certain medical condition would vary and depend on the clinical judgment of each hospital team. Also, surgery is not a treatment option in certain areas of medicine.

### Analytical Methods

A multivariate logistic regression analysis was performed to predict in-hospital mortality by using the development dataset. Tests of model performance and model fitness were conducted using the test dataset. The prediction accuracy of the logistic models was determined using the c-index [25], and the c-index of the full (models 1 and 2) and partial models were compared. A c-index value of 0.5 indicates that the model is no better than random chance in predicting death, and a value of 1.0 suggests perfect discrimination. The models were calibrated by plotting observed versus predicted deaths based on risk. All analyses were conducted with SPSS version 15.0J (SPSS Japan, Inc).

## Results

### Patient Demographics in the Models

Table 1 shows in-hospital mortality by MDCs in the original full dataset. We excluded 6 out of 15 diagnostic categories due to low mortality rates (< 0.5%). The 9 remaining diagnostic categories (n = 224,207) accounted for almost 99% of in-hospital mortality in total acute hospitalization cases. We further grouped 4 MDCs with lowest mortality into one, resulting in 6 MDCs for the following analysis.

**Table 1:** Discharge mortality rate in each Major Diagnostic Categories (n = 282064)

	number of patients	discharge mortality	Discharge moratality rate (%)	Contributing proportion to all discharge mortality (n = 6117)	cumluative moratlity rate	MDC code	category in prediction models
Digestive System, CHepatobiliary System And Pancreas	51514	1932	3.8	31.6	31.6	6	
Respiratory System	30283	1719	5.7	28.1	59.7	4	
Blood and Blood Forming Organs and Immunological Disorders	6070	592	9.8	9.7	69.4	13	
Kidney, Urinary Tract and Male Reproductive System	24415	417	1.7	6.8	76.2	11	others
Nervous System	16192	360	2.2	5.9	82.1	1	
Circulatory System	29408	282	1.0	4.6	86.7	5	
Female Reproductive System, Pregnancy, Childbirth And Puerperium	25659	246	1.0	4.0	90.7	12	others
Injuries, Burns and Others	19113	206	1.1	3.4	94.1	16	others
Breast	4752	151	3.2	2.5	96.6	9	others
Musculoskeletal System And Connective Tissue	16801	142	0.8	2.3	98.9	7	others
Endocrine, Nutritional And Metabolic System	10828	47	0.4	0.8	99.6	10	excluded
Skin, Subcutaneous Tissue	4458	9	0.2	0.1	99.8	8	excluded
Ear, Nose, Mouth And Throat	14086	5	0.04	0.1	99.9	3	excluded
Pediatric disease	3497	5	0.14	0.1	100.0	15	excluded
Eye	19768	3	0.02	0.0	100.0	2	excluded
Newborn And Other Neonates (Perinatal Period)	5220	1	0.02	0.0	100.0	14	excluded

Total	282064	6117	2.2				

Of the 179,156 patients included in the development dataset, 53.2% were male, 35.9% had emergency status at admission, and 8.9% used an ambulance (Table 2). Nearly half (46.6%) of the patients were under 60 years of age at admission, and 9.2% were 80 years or over. The digestive system, hepatobiliary system, and pancreas made up the largest share (22%) of MDCs, followed by the respiratory system (13.5%), circulatory system (13.1%), and nervous system (7.2%). The majority of patients (68.6%) had a total score of 0 for the Charlson Comorbidity Index, and only 2.5% of patients had a score higher than 6.

**Table 2:** Characteristics of patients in learning dataset and test dataset (n = 224207)

		All patients (n = 224207)		Learning dataset (n = 179156)		Test dataset (n = 45051)	
		Patients	%	Patients	%	Patients	%
Major Diagnostic Category	Nervous System	16192	7.2	12996	7.3	3196	7.1
	Respiratory System	30284	13.5	24277	13.6	6007	13.3
	Circulatory System	29407	13.1	23570	13.2	5837	13.0
	Digestive System, CHepatobiliary System And Pancreas	51514	23.0	41125	23.0	10389	23.1
	Blood and Blood Forming Organs and Immunological Disorders	6070	2.7	4829	2.7	1241	2.8
	Others	90740	40.5	72359	40.4	18381	40.8
Sex	male	119216	53.2	95343	53.2	23873	53.0
Age (years)	under60	104341	46.5	83518	46.6	20823	46.2
	60–69	47703	21.3	38148	21.3	9555	21.2
	70–79	51481	23.0	41104	22.9	10377	23.0
	80–89	18033	8.0	14305	8.0	3728	8.3
	90-	2649	1.2	2081	1.2	568	1.3
Status emergency		80515	35.9	64282	35.9	16233	36.0
Use of an ambulance		20052	8.9	15996	8.9	4056	9.0
Total score of Charlson Index	score0	153710	68.6	122898	68.6	30812	68.4
	score1,2	50996	22.7	40812	22.8	10184	22.6
	score3–6	13742	6.1	10856	6.1	2886	6.4
	score7–12	4095	1.8	3234	1.8	861	1.9
	score13-	1664	0.7	1356	0.8	308	0.7
Length of stay (days)	under10	109769	49.0	87979	49.1	21790	48.4
	10–19	52871	23.6	42114	23.5	10757	23.9
	20–29	26190	11.7	20824	11.6	5366	11.9
	30-	35377	15.8	28239	15.8	7138	15.8
Hospital mortality		6047	2.7	4804	2.7	1243	2.8

### Prediction Models (development dataset; n = 179,156)

Table 3 shows the in-hospital mortality prediction model with LOS as a predictor (Model 1). Using those with a LOS under 10 days as a reference, the odds ratio of in-hospital death for patients with longer LOS increased linearly; the odds ratio for patients with LOS ≥ 30 days reached 4.35 (4.01–4.72). Using the neurological MDC as a reference, MDCs for respiratory, digestive, hepatology, and hematology diseases showed a significantly higher odds ratio for in-hospital death, whereas the cardiology MDC showed a significantly lower odds ratio. Older age, gender, use of an ambulance at admission, and emergency admission status also showed significantly higher odds ratios. Finally, scores for Charlson Index categories exhibited an increasing linear trend in odds ratio as scores increased.

**Table 3:** MODEL1 Hospital mortality prediction model with length of stay (n = 179156)

		odds ratio	95% CI		p
			lower	upper	
Sex	Male	1.20	1.13	1.28	0.00
Age	under60, 60–69, 70–79, 80–89, 90-	1.47	1.43	1.51	0.00
Major Diagnostic Category	Nervous System	*			0.00
	Respiratory System	3.97	3.47	4.55	0.00
	Circulatory System	0.69	0.58	0.83	0.00
	Digestive System, CHepatobiliary System And Pancreas	3.27	2.85	3.74	0.00
	Blood and Blood Forming Organs and Immunological Disorders	6.77	5.73	7.98	0.00
	Others	1.27	1.10	1.46	0.05
Status emergency		3.72	3.47	3.99	0.00
Use of an ambulance		1.82	1.68	1.98	0.00
Total score of Charlson Index	score0	*			0.00
	score1,2	1.44	1.33	1.57	0.00
	score3–6	4.07	3.72	4.45	0.00
	score7–12	8.25	7.32	9.29	0.00
	score13-	15.05	12.86	17.61	0.00
Length of Stay	under10	*			0.00
	10–19	1.39	1.26	1.52	0.00
	20–29	1.90	1.71	2.11	0.00
	30-	4.35	4.01	4.72	0.00

Table 4 shows the prediction model without LOS (model 2). The overall statistical significance of odds ratios was completely identical to that of model 1, although the magnitude was somewhat smaller for MDCs and larger for Charlson Index categories.

**Table 4:** MODEL2 Hospital mortality prediction model without length of stay (n = 179156)

		odds ratio	95% CI		p
			lower	upper	
Sex	Male	1.19	1.12	1.26	0.00
Age	under60, 60–69, 70–79, 80–89, 90-	1.58	1.54	1.62	0.00
Major Diagnostic Category	Nervous System	*			0.00
	Respiratory System	3.40	2.98	3.89	0.00
	Circulatory System	0.56	0.47	0.67	0.00
	Digestive System, CHepatobiliary System And Pancreas	2.66	2.32	3.03	0.00
	Blood and Blood Forming Organs and Immunological Disorders	8.09	6.88	9.52	0.00
	Others	1.15	1.00	1.32	0.05
Status emergency		3.72	3.51	3.27	3.76
Use of an ambulance		1.82	1.87	1.72	2.03
Total score of harlson Index	score0	*			0.00
	score1,2	1.63	1.50	1.77	0.00
	score3–6	5.30	4.86	5.77	0.00
	score7–12	10.89	9.70	12.23	0.00
	score13-	19.65	16.87	22.90	0.00

### Model Performance (test dataset; n = 45,051)

Table 2 compares patient characteristics in the test dataset (n = 45,051 patients) to those of the development dataset. The two datasets were almost identical in the distribution of patient characteristics and case mix. In-hospital mortality rates were 2.68% and 2.76% for the development and test datasets, respectively.

**Figure 1:**
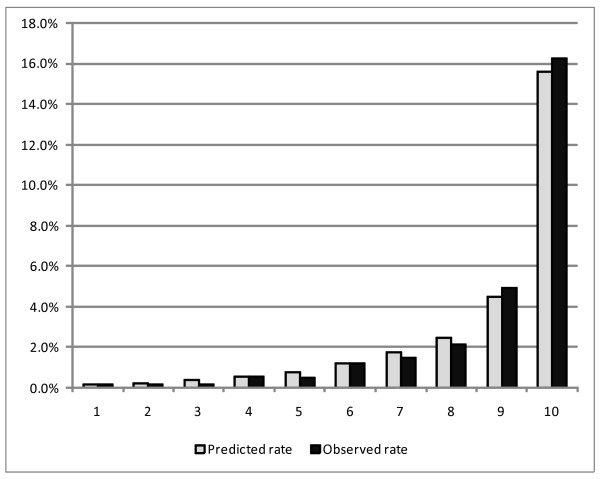
**Model1 hospital mortality prediction model calibration (n = 45051)**. * Figure 1 shows the result of the goodness of fit test regarding the model 1 based on test dataset (n = 45051).

**Figure 2:**
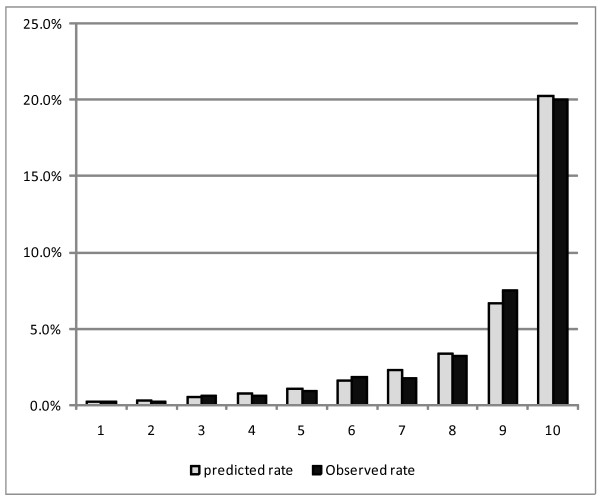
**Model2 hospital mortality prediction model calibration(n = 45051)**. * Figure 2 shows the result of the goodness of fit test regarding the model 2 based on test dataset (n = 45051).

Table 5 shows the c-indexes for models 1 and 2, and those using a partial set of predictors. C-index values were fairly high in both models (0.841 and 0.869 for models 1 and 2, respectively). A partial model which only included patient characteristics had a c-index of 0.727, and the addition of MDC increased the c-index to 0.786. Further including the comorbidity index resulted in only a marginal increase to 0.841. The model that included more information on comorbidities showed a higher c-index. Figures 1 and 2 demonstrate the goodness of fit regarding the models (i.e., how well the predicted mortality rates match the observed mortality rates among patient subgroups of risk). Close agreement between the predicted and observed mortality rates with our models was seen across various patient risk subgroups analyzed.

**Table 5:** Hospital mortality prediction model performance metrics

	C-index (95%CI)
Model 1 (with length of hospital stay)	0.869 (0.860–0.879)
Model 2 (without length of hospital stay)	0.841 (0.830–0.852)
Patients characteristics only (age, sex, status emergency, use of an ambulance)	0.727 (0.713–0.742)
Patients characteristics with MDC	0.786 (0.773–0.799)
Charlson Index only main diagnosis	0.585 (0.567–0.603)
Charlson Index with up to 4 diagnosis	0.639 (0.621–0.692)
Charlson Index with up to 6 diagnosis	0.675 (0.657–0.692)

## Discussion

The prediction model of in-hospital mortality developed in this study is fairly consistent with observed mortality. Results also suggest that inclusion of both comorbidity and other demographic/clinical characteristics of patients account for the better performance of our model compared to a previously described model [17]. When administrative data are used in clinical outcomes research, algorithms to code comorbidities are essential for defining comorbidities. Charlson comorbidity measurement tools [16] are widely used with administrative data to determine the burden of the disease or case-mix. Past studies suggest that the original Charlson Index by chart review and its adaptations for use with administrative databases discriminate mortality similarly [15,17]. The database used in this study assigns to each patient one to six diagnostic codes. Counting multiple comorbidities markedly enhanced accuracy compared to counting comorbidity based on a single ICD-10 code. In addition to comorbidities based on ICD-10 codes, MDCs were also incorporated into our models. By including MDCs, our model could better reflect the characteristics of major patient conditions among all co-existing diagnoses. This may also help to explain the improved performance of our model compared to former prediction models (c-index: 0.69–0.71) which incorporated only the Charlson Index in the analysis of Japanese data [17].

Recent studies in the U.S. introduced a new risk prediction model that includes extended administrative data with lab test results [30,31]. Although the inclusion of detailed clinical data may further improve prediction performance, it requires a sophisticated standardized information system on a nationwide scale. Our prediction model exhibited a comparable level of precision, using variables easily accessible in conventional administrative electronic record systems. As we demonstrated, inclusion of patient demographics, conditions at admission, and the category of major diagnosis with a summary score of comorbidities may be useful and efficient in improving model performance.

In the present study, we developed two models that include and exclude LOS. It is possible that a hospital may promote premature discharge in order to lower in-hospital mortality, thereby adjusting for LOS to allow for a fair comparison of hospital performance. However, the duration of hospitalization is a parameter reflecting various factors other than in-hospital mortality risk, such as the quality of hospital management and socio-economic conditions that facilitate earlier discharge (e.g. availability of informal care at home). Since no major difference in accuracy was observed between the two models, we believe that the use of model 1, which excludes LOS, would be more suitable to adjust for the likelihood of in-hospital death purely due to patient conditions.

In contrast to the risk factor of age, gender did not have a pronounced impact on mortality in our study. Previous studies on cardiovascular surgery in Japan have also shown that the impact of gender on in-hospital mortality is negligible even in risk prediction models with detailed clinical variables [9]. The odds ratio of the circulatory system category was unexpectedly low and may require some explanation. The average risk of cardiovascular hospitalization may have been relatively low in this study because many patients are hospitalized for cardiac catheterization as a post-intervention evaluation in Japan. Thus, an alternative model that categorizes hospitalization for evaluation separately may increase performance in Japanese cases and deserves further consideration in future studies.

A number of limitations of this study are worth noting. Exclusion of 6 low mortality MDCs might bias the performance of our models. Given the c-index for model 2 (n = 282,064) was 0.854, we believe that our model can be useful for hospital mortality analysis in all types of disease. Nevertheless, it would be necessary to update the hospital prediction model periodically, given that the relative importance of factors contributing to mortality may change due to future medical innovations in diagnosis and therapy.

## Conclusion

This study is one of the few Japanese studies that verifies and demonstrates the accuracy of in-hospital mortality prediction models that take into account all diseases. As standardized hospital mortality rates could be used as indicators of quality of care and in setting national standards, risk adjustment in relation to in-hospital mortality is thought to be useful in implementing hospital-based efforts aimed at improving the quality of medical treatment[26]. The risk model described in this study demonstrates a good degree of discrimination and calibration. In addition to its statistical evaluation, it is important that the model can be readily used for risk prediction by clinicians in the field. A major task for the future is to consider how to improve this model in order to make it more detailed, its analytical qualities even more convincing, and its use more compelling.

## Competing interests

The authors declare that they have no competing interests.

## Authors' contributions

HM conceived of the study and designed the protocol. HM and HH1 wrote the paper. HH2 managed data collection and data cleaning. All authors have read and approved the final manuscript.

## Pre-publication history

The pre-publication history for this paper can be accessed here:


